# Diagnosis of pulmonary arterial hypertension: a statement from the Brazilian Thoracic Association

**DOI:** 10.36416/1806-3756/e20250065

**Published:** 2026-03-05

**Authors:** Jose Leonidas Alves-Jr, Veronica Moreira Amado, Ricardo Amorim Correa, Frederico A F Thadeu Campos, Caio Fernandes, Eloara V M Ferreira, Marcelo B Gazzana, Marcelo Jorge Jacó Rocha, Carlos Jardim, Jaquelina S Ota-Arakaki, Virginia Pacheco Guimarães, Monica Corso Pereira, Roberta P Ramos, William Salibe-Filho, Rogerio Souza, Daniel Waetge, Rudolf K F Oliveira

**Affiliations:** 1. Divisao de Pneumologia, Instituto do Coracao, Hospital das Clinicas - HCFMUSP - Faculdade de Medicina, Universidade de Sao Paulo, Sao Paulo (SP) Brasil.; 2. Faculdade de Medicina, Universidade de Brasília, Campus Darcy Ribeiro, Brasília (DF) Brasil.; 3. Divisão de Medicina Interna/Pneumologia, Faculdade de Medicina, Universidade Federal de Minas Gerais - UFMG - Belo Horizonte (MG) Brasil.; 4. Instituto Pequenas Missionarias de Maria Imaculada, Hospital Madre Teresa, Belo Horizonte (MG) Brasil.; 5. Disciplina de Pneumologia, Departamento de Medicina, Universidade Federal de São Paulo - UNIFESP - São Paulo (SP) Brasil.; 6. Serviço de Pneumologia, Hospital de Clínicas de Porto Alegre - HCPA - Universidade Federal do Rio Grande do Sul - UFRGS - Porto Alegre (RS) Brasil.; 7. Hospital de Messejana, Fortaleza (CE) Brasil.; 8. Disciplina de Pneumologia, Departamento de Clínica Médica, Faculdade de Ciências Médicas, Universidade Estadual de Campinas - UNICAMP - Campinas (SP) Brasil.; 9. Divisão de Pneumologia, Departamento de Medicina Interna, Universidade Federal do Rio de Janeiro - UFRJ - Rio de Janeiro (RJ) Brasil.

**Keywords:** Hypertension, pulmonary, Cardiac catheterization, Hemodynamics, Diagnostic techniques, cardiovascular, Risk assessment

## Abstract

Pulmonary arterial hypertension (PAH) is a condition that predominantly affects the pulmonary arterial bed, leading to pulmonary vascular remodeling, progressive decrease in pulmonary arterial compliance, and increase in pulmonary vascular resistance. The symptoms of PAH are nonspecific, which often contributes to diagnostic challenges and significant delays in establishing the diagnosis. The investigation of PAH is extensive and involves thorough search for potential clinical conditions that may contribute to its development, as well as the exclusion of other causes of pulmonary hypertension. The proper hemodynamic definition of PAH requires right heart catheterization (RHC) and direct measurements of mean pulmonary artery pressure, pulmonary arterial wedge pressure, cardiac output, and pulmonary vascular resistance. Additionally, RHC allows the assessment of the severity of the disease and, in selected cases, the identification of patients with a positive pulmonary vascular vasoreactivity test. RHC also provides valuable longitudinal information for risk stratification and patient follow-up. In the current manuscript, we review the PAH diagnostic workup, including a detailed review of the most up-to-date recommendations for right RHC and patient risk stratification.

## INTRODUCTION

Pulmonary hypertension (PH) is a hemodynamic condition of abnormal elevation of pulmonary artery pressure that can occur in association with different diseases. This diagnostic review was conducted as a narrative review by experts in the field of pulmonary hypertension, based on critical appraisal of the available literature and clinical experience. The current PH classification is structured into five groups, according to similarities in pathophysiological mechanisms, clinical and hemodynamic presentations, and, especially, therapeutic approach (Chart 1).^1^ Pulmonary arterial hypertension (PAH) is classified as Group 1 PH and is a condition that predominantly affects the pulmonary arterial bed, leading to pulmonary vascular remodeling, progressive decrease in pulmonary arterial compliance (PAC), and increase in pulmonary vascular resistance (PVR).

**Chart 1:** Classification of pulmonary hypertension.^a^

1. Pulmonary arterial hypertension 1.1. Idiopathic 1.1.1. Long-term responders to calcium channel blockers 1.2. Heritable 1.3. Associated with drugs and toxins 1.4. Associated with: 1.4.1. Connective tissue disease 1.4.2. HIV infection 1.4.3. Portal hypertension 1.4.4. Congenital heart diseases 1.4.5. Schistosomiasis 1.5. PAH with features of venous/capillary (PVOD/PCH) involvement 1.6. Persistent PH of the nborn
2. Pulmonary hypertension due to left heart disease 2.1. Heart failure: 2.1.1. Preserved ejection fraction 2.1.2. Reduced or mildly reduced ejection fraction 2.1.3. Cardiomyopathies with specific etiologies 2.2. Valvular heart disease 2.3. Congenital/acquired cardiovascular conditions leading to post-capillary PH
3. Pulmonary hypertension due to lung diseases and/or hypoxia 3.1. COPD and/or emphysema 3.2. Interstitial lung disease 3.3. Combined pulmonary fibrosis and emphysema 3.4. Other parenchymal lung diseases 3.5. Nonparenchymal restrictive diseases 3.5.1. Hypoventilation syndromes 3.5.2. Pneumonectomy 3.6. Hypoxia without lung disease (e.g., exposure to high altitude) 3.7. Developmental lung diseases
4. Pulmonary hypertension with pulmonary artery obstructions 4.1. Chronic thromboembolic pulmonary hypertension 4.2. Other pulmonary artery obstructions: sarcomas, other tumors, arteritis, congenital pulmonary arterial stenosis, parasites (hydatidosis)
5. Pulmonary hypertension with unclear and/or multifactorial mechanisms 5.1. Hematologic disorders: chronic hemolytic anemia, myeloproliferative disorders, splenectomy 5.2. Systemic disorders: sarcoidosis, pulmonary histiocytosis, neurofibromatosis 5.3. Metabolic disorders: glycogen storage disease, Gaucher disease, thyroid disorders 5.4. Chronic renal failure (with or without hemodialysis) 5.5. Pulmonary tumor thrombotic microangiopathy 5.6. Fibrosing mediastinitis 5.7. Complex congenital heart disease

PVOD: pulmonary veno-occlusive disease; and PCH: pulmonary capillary hemangiomatosis.

a Modified from Kovacs et al.^1^

The incidence and prevalence of PAH are around 6 and 48-55 cases/million population. The female-to-male ratio is about 1.7:1.0. However, there has been a tendency towards a more balanced distribution between genders and an increase in the average age of affected patients in recent years.^2^ ^,^ ^3^ Without treatment, the estimated median survival was reported to be 2.8 years for patients with idiopathic PAH.^4^. Currently, in the modern treatment era, the PAH survival rate at 3 years is around 75%.^5^ ^,^ ^6^

PAH pathophysiology is multifactorial, including genetic predisposition, environmental exposures, and endothelial dysfunction. Histopathological findings show predominant involvement of the distal arterial bed (vessels < 500 µm), but veno-capillary involvement may occur, especially in cases of pulmonary veno-occlusive disease (PVOD), pulmonary capillary hemangiomatosis (PCH), and PAH associated with scleroderma. Arteriolar lesions are characterized by medial hypertrophy, intimal proliferation and fibrosis, and adventitial thickening. In addition to vascular remodeling, it is common to identify perivascular inflammatory infiltrate and thrombotic lesions, which indicate the pathophysiological involvement of inflammatory pathways and the interaction between the endothelial lesion and the coagulation/fibrinolysis system, favoring the formation of thrombi in the pulmonary microcirculation.^7^ Endothelial dysfunction participates in vascular remodeling through different and complex pathways including: (1) an increase in vasoconstrictor agents predisposing to tissue proliferation, such as thromboxane A2 and endothelin, and reduction in vasodilators and antiproliferative agents, such as nitric oxide and prostacyclin; (2) production of growth factors such as VEGF and PDGF ; and (3) reduction or dysfunction of bone morphogenetic protein receptor type 2 (BMPR2)-a receptor in the TGF-β superfamily involved in regulating cell proliferation and apoptosis-that has been implicated in both hereditary and acquired forms of PAH. On the other hand, the significant vasoconstriction observed in patients who are considered responders to the acute vasoreactivity test is also influenced by changes in ion channels in smooth muscle cells.^7^

Pulmonary vascular remodeling, arteriolar thrombosis, and vasoconstriction determine a progressive decrease in PAC and subsequent increase in PVR. In response to the increase in cardiac afterload, the right ventricle (RV) hypertrophies and dilates, progressing to ventricular-vascular uncoupling and subsequent RV failure. RV dysfunction is associated with poor clinical outcomes and is the leading cause of death in PAH patients.^8^ ^,^ ^9^

The diagnosis of PAH requires right heart catheterization (RHC) and direct measurements of mean pulmonary artery pressure (mPAP), pulmonary arterial wedge pressure (PAWP), cardiac output (CO), and PVR. The hemodynamic definition of PAH has undergone recent modifications. It has moved from being an empirical criterion to a definition based on pulmonary hemodynamic data derived from normal individuals, which has prognostic relevance. Thus, the current diagnostic criterion for resting PH is a mPAP > 20mmHg, since a normal mPAP is around 14 mmHg, and considering two standard deviations above this level as the limit of normality.^9^ ^-^ ^11^ To characterize pre-capillary PH, the following criteria must be met at rest:^9^

- mPAP > 20 mmHg
- PAWP or left ventricular end-diastolic pressure (LVEDP) ≤ 15 mmHg, and
- PVR > 2.0 Wood units (or > 160 dyn *·s·* cm^-^⁵)

## CLINICAL PRESENTATION

The symptoms of PH (dyspnea on exertion, palpitation, presyncope or syncope, and chest pain) are nonspecific, which makes PH diagnosis difficult and often leads to delay in diagnosis. On physical examination, the most common findings are hyperphonesis of the pulmonary component of the second heart sound, presence of a systolic murmur characteristic of presence of tricuspid insufficiency, in addition to peripheral edema, jugular swelling, and painful hepatomegaly, demonstrating the presence of right heart failure.^12^

Due to the low specificity in the signs and symptoms associated with PAH, the time between the onset of symptoms and diagnosis is still of approximately two years, often resulting in diagnosing the disease in more advanced stages, precluding early therapeutic interventions.^13^ ^,^ ^14^

When assessing dyspnea in patients with PAH, the use of the functional classification of the New York Heart Association (NYHA) modified for PH is recommended, ranging from class I (with no limitation of usual physical activities) to class IV (dyspnea at rest or syncope on exertion).^15^ Despite the subjectivity associated with this classification system, it remains one of the most important prognostic markers in patients with PAH.^9^

The diagnostic investigation of PAH is extensive and demands a systematic search for underlying clinical conditions that may contribute to its development, along with the exclusion of other causes of PH. This process benefits from a multidisciplinary perspective-including cardiology, pulmonology, and rheumatology-to ensure a comprehensive evaluation.^9^ ^,^ ^12^ ^,^ ^16^ Below, we present the set of tests associated with the investigation of PH.

## CHEST RADIOGRAPHY

**Figure 1:**
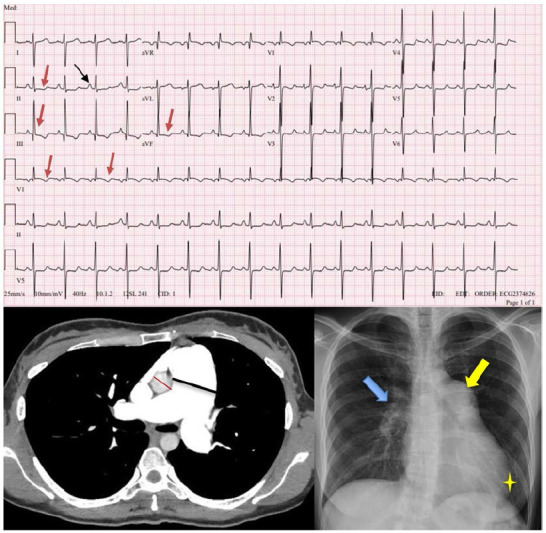
Electrocardiogram, chest CT, and chest radiograph in pulmonary hypertension. Three tests were conducted to investigate pulmonary hypertension in a 32-year-old female patient diagnosed with PAH. ECG: right ventricular hypertrophy with right axis deviation, P pulmonale (black arrow), and right ventricular strain pattern (ST depression in leads II, III, and aVF and T inversion in V1-2, red arrows). Chest contrast-enhanced CT: pulmonary artery enlargement (> 29 mm - black line) and a pulmonary artery/aorta ratio greater than 1.0 (aorta diameter - red line). Chest radiograph (frontal): cardiomegaly with an elevated cardiac apex (yellow star), prominent pulmonary trunk (yellow arrow), and enlarged pulmonary arteries (blue arrow). PAH: pulmonary arterial hypertension; and ECG: electrocardiogram.

Although chest radiography is an inexpensive and widely available test, it is not useful in the initial stages of PH. However, it can serve as a screening tool for more severe cases (Figure 1). Signs of parenchymal lung disease may be found in this exam, suggesting group 3 PH or enlarged left heart chambers with pulmonary congestion, suggesting group 2 PH. Findings of enlargement of the right atrium (RA) and RV, enlargement of the right interlobar artery (> 15 mm in diameter), hilar enlargement, and areas of oligoemia may suggest group 1 or 4 PH.^17^

## ELECTROCARDIOGRAM

Commonly, electrocardiogram (ECG) shows signs of right chamber dilatation and hypertrophy-axis deviation to the right, P pulmonale wave (P > 2.5 mm in DII) and right ventricular strain pattern characterized by ST depression, and T wave inversion in right ventricular leads (V1-4, II, III and aVF). However, these findings are observed in the phases when cardiac repercussion of the vascular involvement is unequivocal (Figure 1). In up to 13% of cases, ECG is reported as normal.^18^ ECG might also help identifying indicators of left-sided heart disorders, assisting therefore in narrowing down the differential diagnosis.

## CHEST CT

**Figure 2:**
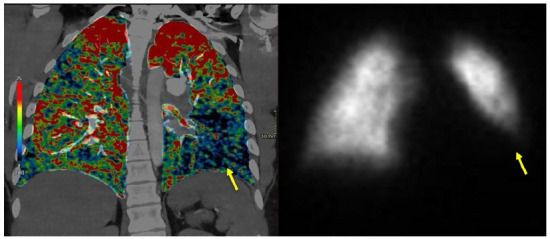
Dual-energy CT of the chest and lung perfusion scintigraphy. Dual-energy CT iodine map and lung perfusion scintigraphy used to investigate pulmonary hypertension. In this case, the two diagnostic methods agree and show hypoperfusion in the left lower lobe (yellow arrow).

The measurement of the pulmonary artery has high specificity for detecting PH when the diameter is greater than 29 mm and the pulmonary artery/aorta ratio > 1.0 (Figure 1).^19^ HRCT is an important test to detect patients in group 3 PH. The presence of mosaic is more characteristic of chronic thromboembolic pulmonary hypertension (CTEPH), and contrast-enhanced CT can detect thrombi or other differential diagnoses of group 4 PH. Contrast-enhanced CT is not the gold standard for ruling out group 4 PH, but dual-energy CT (Figure 2) has appeared as a promising substitute for V/Q scintigraphy. CT also plays a fundamental role in raising the suspicion of the presence of a significant veno-occlusive component when there are septal lines, centrilobular ground-glass opacities, and lymphadenopathy, as well as in ruling out the presence of significant parenchymal lung disease.^20^

## ECHOCARDIOGRAPHY

**Figure 3:**
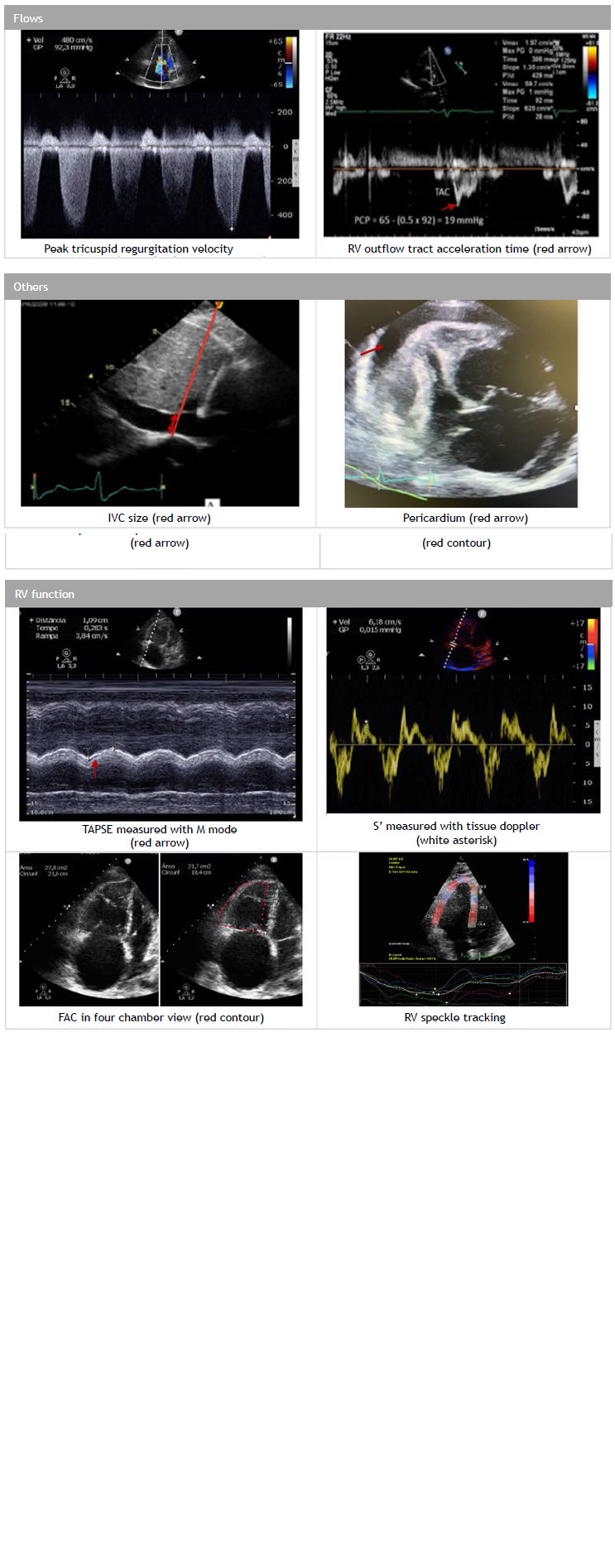
Echocardiogram parameters in pulmonary hypertension. RV: right ventricle; LV: left ventricle; RA: right atrium; TAPSE: tricuspid annular plane systolic excursion; S’: peak systolic velocity of tricuspid annulus; FAC: fractional area change; and IVC: inferior vena cava.

Echocardiography is the most important noninvasive screening tool for PH, being able to provide information on chamber size, function, and flows, particularly for parameters related to the right heart (Figure 3). However, it is important to note that echocardiography has moderate precision in accurately estimating pulmonary artery pressures, having the potential for both overestimation and underestimation. Therefore, it does not allow for a definitive diagnosis or a clear differentiation between the distinct groups of PH and confirmatory invasive testing remains mandatory for PH diagnosis and hemodynamic classification.

Echocardiography detects indirect signs of increased pulmonary pressure and estimates the systolic pulmonary artery pressure (sPAP) based on the pressure gradient between the RV and the RA during systole. This is calculated using the maximum velocity of the tricuspid regurgitation jet (TRJV) and an estimation of right atrial pressure. A TRJV exceeding 3.4 m • s^-^¹ strongly indicates a high probability of PH, regardless of other echocardiographic findings. Although a TRJV between 2.9 and 3.4 m • s^-^¹ indicates an intermediate probability of PH-and values of 2.8 m • s^-^¹ or lower generally suggest low probability-this assessment can be significantly influenced by additional echocardiographic findings, particularly morphological changes and function aspects of the RV that support a higher probability of PH.

Due to the anatomical complexity of the RV, multiple indices are employed for quantitative assessment, in addition to qualitative evaluation of the chamber:

- Morphological parameters include RV enlargement, flattening of the interventricular septum (assessed by the eccentricity index), increased pulmonary artery diameter and flow, enlargement of the right atrial area, and changes in the diameter and respiratory variability of the inferior vena cava.^21^
- Functional parameters involve several echocardiographic measures: tricuspid annular plane systolic excursion (TAPSE); fractional area change (FAC), which compares the RV area between systole and diastole; myocardial performance index (MPI); and right ventricular ejection fraction (RVEF), assessed using both two- and three-dimensional modalities. Additional indicators include the systolic velocity of the lateral tricuspid annulus (S′ wave), obtained through tissue Doppler imaging, and RV longitudinal strain, which can be evaluated globally or specifically in the free wall.^22^ ^-^ ^24^
- RV and pulmonary artery coupling refer to the interaction between RV contractility and afterload. This relationship, known as ventriculoarterial coupling, can be estimated using the TAPSE/sPAP ratio, a parameter recognized as a prognostic marker in PAH.^25^

## CARDIAC MRI

Cardiac MRI (CMRI) can provide information on RV mass and changes in mass, RV volumes and changes in volume, RV function and relation to other markers, RV damage, and RV/left ventricle (LV) interaction. Therefore, CMRI has the potential to provide an integrated functional and structural assessment of the cardiopulmonary system in a single exam that is established as valuable prognostic markers in PH. The main limitation for routine use of CMRI is its low availability and high cost. Currently, right ventricular end-systolic volume index (RVEF) and stroke volume index are the strongest prognostic parameters of PAH on CMRI.^9^ ^,^ ^26^ ^,^ ^27^ Additionally, CMRI can show signs of congenital heart disease and left heart disease.

## PULMONARY FUNCTION TEST

Pulmonary function test (PFT) is an important exam not only to differentiate Group 3 PH but also plays a role in identifying comorbidities given the high prevalence of obstructive diseases in the general population and the relevance of restrictive diseases in the autoimmune context. DL_CO_ may be reduced and may be related to prognosis in PAH. DL_CO_ should be part of the follow-up of patients with systemic sclerosis in order to increase suspicion of pulmonary vascular involvement.^28^ ^,^ ^29^

## V/Q SCINTIGRAPHY

V/Q scintigraphy is the exam of choice to rule out CTEPH in the diagnostic evaluation of PH, either planar or single-photon emission CT with less than two segmental mismatched perfusion defects.^30^ Perfusion scintigraphy can reveal changes in PAH with heterogeneity in lung perfusion, and a patchy pattern of lung perfusion defect is commonly seen. Patients with a patchy pattern identified by lung perfusion scintigraphy were associated with more severe disease and worse outcome.^31^

## BLOOD TESTS

The evaluation of blood tests is important in the diagnosis, whether in differentiating PH groups or PAH subtypes, and in the prognosis of PAH. The evaluation involves blood count, liver and kidney evaluation, thyroid function, and BNP or NT-proBNP. Arterial blood gas analysis could be valuable. A severe reduction of PaO_2_ in PAH might raise suspicion for patent *foramen ovale*, other causes of shunt or low-DL_CO_-associated conditions. Furthermore, low PaCO_2_ is a prognostic marker, and high PaCO_2_ could indicate hypoventilation, which in itself may be a cause of PH.^9^

## EXERCISE TESTING

Cardiopulmonary exercise testing (CPET) provides information on gas exchange, ventilatory efficacy, and cardiac function during exercise. Thus, it offers a pathophysiological evaluation of exercise limitations and dyspnea of patients. In PH, CPET may present both circulatory impairment and ventilatory inefficiency, characterized by hyperventilation, low end-tidal partial pressure of carbon dioxide (PetCO_2_), high ventilatory equivalents for oxygen and carbon dioxide, elevated ventilation/carbon dioxide production (V_E_/VCO_2_) slope and low O_2_ pulse. Some CPET parameters have shown significant prognostic value in PAH. O_2_ consumption at exercise peak (peak VO_2_) and V_E_/VCO_2_ slope are currently included among the markers suggested by European guidelines for risk stratification in PAH patients.^9^ ^,^ ^32^ ^-^ ^34^ The six-minute walk test is useful for risk stratification and evaluation of effectiveness of therapy.

## RHC

RHC is the gold standard procedure for the diagnosis and the hemodynamic classification of PH.^9^ Additionally, RHC is required to assess the severity of pulmonary vascular and RV impairment and to perform vasoreactivity testing of the pulmonary circulation, when appropriate.^35^ Furthermore, beyond its role in PH diagnosis, RHC has a major role in patient follow-up, providing valuable longitudinal information on disease severity and response to specific PAH therapy.^36^

The execution of RHC demands not only technical expertise but also strict adherence to standardized methodology.^9^ Failure to follow best practices can easily lead to inaccurate hemodynamic measurements and misdiagnosis. To ensure reliable results and minimize risks, RHC should be performed exclusively in expert centers, where trained professionals can maintain procedural accuracy and patient safety.^35^ In such settings, the incidence of severe adverse events is reported to be below 1.1%.^37^ Rigorous training is essential to avoid misinterpretation of pressure waveforms and data, which may compromise clinical decision-making. Contraindications to RHC include the presence of a right heart tumor or thrombus, a mechanical tricuspid valve, a pacemaker implanted within the previous month, and/or active infection.

**Figure 4:**
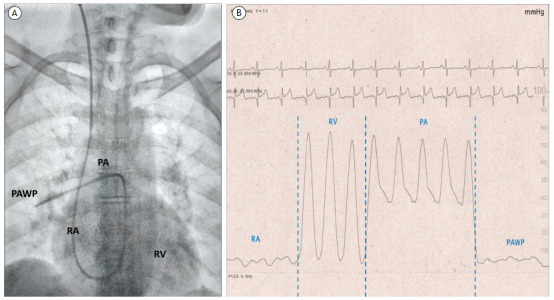
(A) Fluoroscopy image performed during a right heart catheterization. (B) Graphic representation of the pressure waveforms. RA: right atrium; RV: right ventricle; PA: pulmonary artery; and PAWP: pulmonary arterial wedge pressure.

Prior to performing RHC, pre-existing medical conditions must be optimized, especially systemic blood pressure and volemia. The appropriate preparation of patients for RHC is of key relevance and can majorly impact the patient’s safety, hemodynamic results, and treatment decisions.^9^ RHC should be performed at rest in the supine position, in a hemodynamic room, and guided by ultrasonography and by fluoroscopy (Figure 4). RHC is preferably performed using a flow-directed pulmonary-artery catheter, also known as the Swan-Ganz catheter.^38^ In this case, the venous access can be via the internal jugular vein, brachial vein, or femoral vein. Due to anatomical reasons, the right internal jugular vein is the first choice in most expert centers when using Swan-Ganz catheters.

During an RHC, there are common sources of errors that can substantially influence hemodynamic measurements.^39^ Among them, the incorrect determination of the zero-reference level is the most frequent one.^9^ Current evidence indicates that, in the supine position, the zero-reference level should be determined at the mid-thoracic level, which is at the level of the left atrium in most patients.^40^

Invasive pressure measurements should be made in the RA, RV, pulmonary artery (PA), and PA at wedge position (PAWP). Pressure measurements should preferably be obtained through the analysis of the hemodynamic curves, using simultaneous electrocardiographic tracing (Figure 4). Pulmonary pressures can be measured at the end of expiration during continuous breathing or preferably by averaging the values over 3-4 respiratory cycles.^41^ This method is preferred when there is a significant change in intrathoracic pressure during the respiratory cycle (obesity, COPD, and/or during exercise, for example).^42^ PAWP is obtained by inflating the pulmonary artery-catheter distal balloon or impacting the catheter in the distal pulmonary vascular bed. Oxygen saturations obtained with the pulmonary-artery catheter in the wedged position can be used to confirm a precise catheter positioning (saturation > 90% or within 5% of systemic arterial saturation).^43^ If no reliable PAWP curve is acquired or if PAWP is > 11 mmHg,^44^ it is appropriate to consider the measurement of LVEDP or to perform stress testing of the pulmonary vascular system (either fluid challenge or exercise RHC).

CO can be assessed by the direct Fick method (i.e., with simultaneously directly measured oxygen consumption), by thermodilution (using the mean value of three CO measurements within a 10% variance range), or the indirect Fick method (using estimated oxygen consumption). The direct Fick method is the gold standard. The indirect Fick method is less reliable when compared with the thermodilution method,^45^ given that an assumed or estimated oxygen consumption is known to be inaccurate and prone to meaningful error.^39^ Additionally, CO measured by the appropriate thermodilution technique is trustworthy even in the setting of low CO or severe tricuspid regurgitation.^46^ However, the thermodilution technique should not be used in the presence of shunts^47^; in this case, a stepwise assessment of oxygen saturation should be performed.^48^ The same applies when the SvO_2_ is identified to be > 75%.

Derived variables calculated from RHC measurements should include cardiac index (CI), stroke volume (SV), SV index, pulmonary vascular resistance (PVR), PVR index, PAC, systemic vascular resistance (SVR), and SVR index. Charts 2 and 3 summarize the measured and calculated variables obtained during an RHC.

**Chart 2:** Right heart catheterization variables (thermodilution method).

Measured variables
Heart rate, bpm
Systemic blood pressure (SBP): systolic, diastolic, and mean, mmHg
Arterial oxygen saturation (SaO_2_), %
Right atrial pressure (RAP), mean (mmHg)
Right ventricular pressure (RVP), systolic and end-diastolic, mmHg
Pulmonary arterial wedge pressure (PAWP), mean (mmHg)
Pulmonary arterial pressure (PAP), systolic and diastolic, mean (mmHg)
Cardiac output (CO), L/min
Mixed-venous oxygen saturation (SvO_2_), %
Calculated variables
Cardiac index (CI): CO/body surface area, L/min/m^2^
Stroke volume (SV): CO/HR * 1000, mL/beat
Stroke volume index (SVI): (CI / HR) *1000 (mL/beat/m^2^)
Pulmonary vascular resistance (PVR): (mPAP − PAWP) / CO (WU)
Pulmonary vascular resistance index (PVRI): (mPAP − PAWP) / CI (WU/m^2^)
Pulmonary arterial compliance (PAC): (sPAP − dPAP) / SV (mL/mmHg)
Systemic vascular resistance (SVR): (mSBP − RAP) / CO (WU)
Systemic vascular resistance index (SVRI): (mSBP − RAP) / CI (WU/m^2^)

**Chart 3:** Hemodynamic profiles of pulmonary hypertension.

Profile	Hemodynamic characteristics	Associated PH groups
Pre-capillary PH	mPAP > 20 mmHg PAWP ≤ 15 mmHg PVR > 2 WU	I, III, IV, V
Isolated post-capillary PH	mPAP > 20 mmHg PAWP > 15 mmHg PVR < 2 WU	II
Combined post-capillary PH	mPAP > 20mmHg PAWP > 15mmHg PVR > 2 WU	II, V
Exercise PH	mPAP/CO slope > 3mmHg/L/min	I, II, III, IV, V
Exercise postcapillary PH	PAWP/CO slope > 2mmHg/L/min or PAWP > 20-25 mmHg during supine exercise or PAWP > 17-19 mmHg during upright exercise	II

PH: pulmonary hypertension; mPAP: mean pulmonary arterial pressure; PAWP: pulmonary arterial wedge pressure; PVR: pulmonary vascular resistance; CO: cardiac output; HFpEF: heart failure with preserved ejection fraction

After performing an RHC, it is critical to define the hemodynamic profile (Chart 3) to determine the affected vascular territory. Pre-capillary PH is characterized by all the following:

- mPAP > 20 mmHg
- PVR > 2.0 Wood Units (WU)
- PAWP ≤ 15 mmHg

This profile is typically associated with conditions involving pulmonary arterial pathology with no significant left heart disease, such as PAH. In contrast, post-capillary PH, strongly indicating the presence of left heart disease, is defined by:

- mPAP > 20 mmHg
- PAWP > 15 mmHg

Further subclassification is based on PVR values:

- Isolated post-capillary PH is identified by PVR ≤ 2 WU

### Combined pre- and post-capillary PH is distinguished by PVR > 2 WU. Acute vasoreactivity testing

In PAH, vasoreactivity testing identifies acute vasoresponders who may benefit from treatment with high-dose calcium channel blockers. The test should only be performed in idiopathic PAH, hereditary PAH, or drug-induced PAH. Possible acute vasoreactivity testing compounds include inhaled nitric oxide^49^ or inhaled iloprost.^50^ ^,^ ^51^ Inhaled nitric oxide should be administered at 10-20 ppm for 5-10 min. Inhaled iloprost should be administered at 5-10 µg for 10-15 min. Epoprostenol is also a validated option, but not available in Brazil. Acute vasoactive testing is considered positive when all of the following criteria are met: (1) mPAP decrease ≥ 10 mmHg; (2) mPAP (absolute value) ≤ 40 mmHg; and (3) CO without decrease from baseline.^49^ Patients who have a positive result in the vasoreactivity test should initially be treated with calcium channel blockers. However, due to the uncertainty of a long-term response, they should be closely monitored, and in the absence of a satisfactory hemodynamic response, specific medications for the treatment of PAH should be added.^49^

### Fluid challenge

In patients with a clinical suspicion of LV diastolic dysfunction but with a pre-capillary hemodynamic pattern (i.e. PAWP ≤ 15 mmHg), or in patients with a PAWP between 12-15 mmHg,^44^ fluid challenge should be considered to uncover a post-capillary hemodynamic pattern (i.e. occult left heart disease). A proper fluid challenge should be done using 500 mL of saline solution infused at a rate of approximately 100 mL/min,^52^ yielding a total test length of approximately 5 min. The most important aspect of the fluid challenge is the rate of infusion, considering that the cardiopulmonary system can accommodate high increases of blood flow under physiological conditions. A PAWP ≥ 18 mmHg post-fluid challenge suggests LV diastolic dysfunction.^53^

### Exercise RHC

Pulmonary vascular responses to exercise have been recently reintroduced as a criterion for PH,^9^ and exercise RHC is considered the gold standard for the diagnosis of exercise PH.^54^ The association of exercise hemodynamics with CPET and direct blood gas analysis allows the proper identification of the mechanisms of exercise intolerance in PH and other diseases.^55^ A mPAP/CO slope > 3 mmHg/L/min has been proposed as the diagnostic threshold for exercise PH, considering normal resting hemodynamics.^9^ The steep increase of mPAP in relation to CO, with normal left ventricular filling pressures, might suggest early pulmonary vascular remodeling,^56^ which impacts exercise capacity and prognosis. On the other hand, a steep increase of PAWP in relation to CO (> 2 mmHg/L/min), with normal resting PAWP and normal LV systolic function, might suggest heart failure with preserved ejection fraction (HFpEF).^57^ However, an exercise PAWP cut-off of > 20 mmHg or > 25 mmHg during supine exercise, or > 17-19 mmHg during upright exercise has also been proposed for the diagnosis of HFpEF.^58^ ^-^ ^60^ Even though a mPAP/CO > 3 mmHg/L/min is abnormal across all ages, it is important to note that the upper limits of normal pulmonary hemodynamics vary strongly according to age.^61^ ^,^ ^62^

During exercise, pressures must be averaged over 3-4 respiratory cycles. The exercise protocol should be incremental and symptom-limited. Hemodynamic measurements should be assessed at each exercise stage. Beyond PAP (systolic and diastolic), PAWP and CO, RA pressure (RAP), heart rate, and systemic blood pressure should also be measured. Dynamic arterial and mixed-venous oxygen saturation and content should be assessed as well. Calculated hemodynamic variables include total pulmonary resistance, PVR, CI, SVi, mPAP/CO, and PAWP/CO slopes. RAP/CO slope might also be useful to assess RV function. Exercise right ventricular stroke work index is an additional calculated variable that is associated with functional capacity and might help identify abnormal pulmonary vascular responses to exercise.^56^ ^,^ ^63^

Exercise RHC is mostly used for the investigation of dyspnea of unknown origin, when resting RHC is normal.^55^ Nonetheless, exercise hemodynamics can also be useful in patients with established PH for the investigation of unclear pathophysiological mechanisms, access to functional status, response to treatment, and prognosis.^62^ ^-^ ^64^

## ADDITIONAL EVALUATION FOR PH

There are several mutations associated with an increased risk of developing PAH. The most important ones are those related to BMPR2, which can be identified in approximately 80% of patients with familial PAH and in 20% of sporadic PAH.^65^ Genetic evaluation is important for counseling patients and their families, as well as for making early identification of the disease possible. However, genetic evaluation is still restricted to very few centers in the world and is not a reality in the current Brazilian public healthcare system.

Patients diagnosed with PAH must undergo additional laboratory tests for diagnostic characterization. The association with connective tissue diseases (CTD) must be clinically investigated, and autoantibody tests must be guided according to clinical suspicion. In patients who do not present stigmata of CTD, rheumatologic screening should be carried out with assessment of, at least, antinuclear antibodies and serum complement.^66^ Serology for HIV, hepatitis B, and hepatitis C should be performed to rule out the possibility of an association of PAH with HIV and/or chronic liver diseases (with portal hypertension). In cases where liver involvement is suspected, upper abdominal ultrasound can provide valuable information. Abdominal ultrasound is particularly important in patients with an epidemiological history of schistosomiasis, as it can reveal periportal fibrosis. In such cases, stool examination using the Kato-Katz technique is also recommended. The diagnosis of schistosomiasis is based on the presence of periportal fibrosis on abdominal ultrasound, along with the detection of *Schistosoma mansoni* eggs in stool samples or in rectal or liver biopsy specimens.^66^ The association of pulmonary hypertension with hemoglobinopathies and thyroid dysfunction should be evaluated with blood workup, thyroid hormonal levels, and further investigations in specific cases. The association of PAH with some drugs has been described and must be questioned in the clinical history (Chart 4 ).^9^

**Chart 4:** Drugs associated with pulmonary arterial hypertension.

Definite association	Possible association
Aminorex Benfluorex Carfilzomib Dasatinib Dexfenfluramine Fenfluramine Methamphetamines Mitomycin C Toxic rapeseed oil	Alkylating agents (cyclophosphamide, mitomycin C) Amphetamines Bevacizumab Bortezomib Bosutinib Cocaine Diazoxide Direct-acting antiviral agents against hepatitis C virus (sofosbuvir) Indigo naturalis (Chinese herb known as Qing-Dai) Interferon alpha and beta Leflunomide L-tryptophan Phenylpropanolamine Ponatinib Solvents (trichloroethylene) St John’s Wort
Modified from Kovacs et al.^1^	

PVOD and PCH are forms of PH, classified as group 1 PH and with similar characteristics. Despite significant pre-capillary involvement, these diseases generally have a worse prognosis than does idiopathic PAH. Moreover, they are prone to severe pulmonary edema when treated with pulmonary vasodilators, which is why these drugs should be used with great caution in this scenario. Therefore, lung transplantation is the most appropriate alternative to treat these patients. The presence of central lobular ground-glass nodules, enlarged mediastinal lymph nodes, and septal thickening on chest CT of a patient with pre-capillary PH is suggestive of PVOD. Hypoxemia and reduction in DL_CO_ are usually more intense than in other causes of PAH. Bronchoscopy with bronchoalveolar lavage can contribute to the diagnosis when findings of increased hemosiderin-laden macrophages are observed. It should be noted that transbronchial biopsy is prohibited for patients with PAH.^67^ The finding of biallelic mutations in the eukaryotic translation initiation factor 2 alpha kinase 4 (*EIF2AK4*) gene as the cause of heritable PVOD/PCH represents an advance in pathophysiological understanding of these conditions,^68^ but genetic evaluation in not widely available in the current Brazilian public healthcare system.

**Figure 5:**
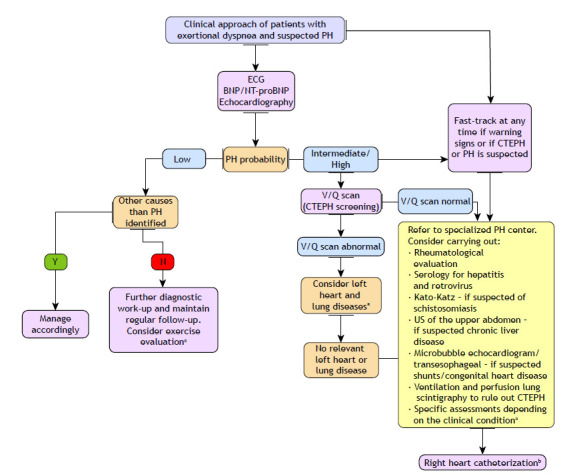
Diagnostic algorithm for pulmonary hypertension. Algorithm for clinical investigation of patients with exertional dyspnea and suspected pulmonary hypertension. BNP/NT-proBNP: brain natriuretic protein/N-terminal pro-brain natriuretic peptide; CTEPH: chronic thromboembolic pulmonary hypertension; ECG: electrocardiogram; PH: pulmonary hypertension; US: ultrasonography; V/Q scan: ventilation/perfusion scintigraphy. ^a^Complementary investigation including pulmonary function tests and cardiopulmonary exercise testing. ^b^Vasoreactivity test for patients suspected of idiopathic, drug-associated, or hereditary pulmonary hypertension; consider volume load test if heart failure with preserved ejection fraction is suspected; exercise test must be considered in some cases. ^c^Exercise assessment can be noninvasive, but in some cases exercise right heart catheterization must be considered.

In cases of unknown etiology, clinical history must be thoroughly reviewed for systemic and other diseases associated with PH (especially Group 5 PH). Below, we present the diagnostic algorithm for PH (Figure 5).

## RISK STRATIFICATION

**Figure 6:**
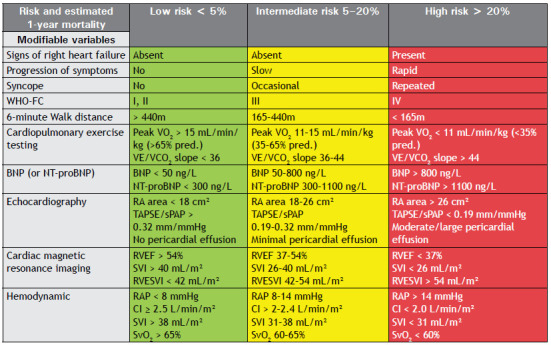
Pulmonary arterial hypertension risk stratification. WHO-FC: World Health Organization functional class; VO_2_: oxygen uptake; pred.: predicted; VE/VCO_2_: ventilatory equivalents for carbon dioxide; BNP: brain natriuretic peptide; NT-proBNP: N-terminal pro-brain natriuretic peptide; RA: right atrium; TAPSE: tricuspid annular plane systolic excursion; sPAP: systolic pulmonary arterial pressure; RVEF: right ventricular ejection fraction; SVI: stroke volume index; RVESVI: right ventricular end-systolic volume index; RAP: right atrial pressure; CI: cardiac index; and SvO_2_: mixed venous oxygen saturation.

Risk stratification of patients with PAH is essential for therapeutic planning, at the time of diagnosis, and during patient follow-up (Figure 6). The multiparametric approach proposed by the ERS/ESC guidelines in 2015 was updated in 2022, maintaining stratification as low, intermediate, and high risk, but including new imaging and hemodynamic parameters, as well as correcting the risk of death in 1 year for moderate-risk stratum (5-20%) and high stratum (> 20%).^9^ Functional class (NYHA), six-minute walk distance, and BNP/NT-proBNP levels reached high predictive values in studies that evaluated risk stratification strategies in PAH.^69^ ^,^ ^70^ Patients can be classified by attributing values of 1 to low risk, 2 to intermediate risk, and 3 to high risk for each variable, according to their values, and calculating the arithmetic mean (the sum of points divided by the number of criteria evaluated). Thus, means between 1.5 and 2.49 indicate intermediate risk, lower means indicate low risk, and higher means indicate high risk.^70^ Another possible approach is to classify patients as low risk (all variables correspond to the proposed limits for low risk) or not low risk (one or more variables are not within the low risk limits).^69^ However, it is essential to include prognostic variables from diagnostic catheterization (RAP, CI, SVi, and SvO_2_) for initial stratification.

Considering that about 60% of PAH patients are classified at an intermediate risk by the strategies that use three strata,^69^ ^,^ ^70^ the intermediate-risk stratum was proposed to be subdivided into low intermediate and high intermediate risk in order to obtain greater sensitivity in detecting patients with a worse prognosis. This classification proved to be more sensitive in detecting changes in risk during patient follow-up. The strata are calculated from the arithmetic mean; thus, means between 1.5 and 1.99 correspond to a low intermediate risk, and those between 2.0 and 2.49 correspond to a high intermediate risk.^71^ Risk re-stratification during follow-up should include at least functional class, natriuretic peptide levels, and 6-minute walk distance. In doubtful cases, other prognostic factors should be evaluated. An alternative approach to risk assessment is the calculator developed from the American registry-Registry to Evaluate Early and Long-Term PAH Disease Management (REVEAL)-which uses 12 variables, or its simplified version REVEAL LITE 2.0, with 6 variables.^72^ ^,^ ^73^ These parameters allow for minimal risk stratification, but additional information from complementary imaging tests and invasive hemodynamics is important throughout the follow-up of these patients.

## PH CENTERS

PAH is a complex disorder needing comprehensive and precise diagnostic evaluation. PAH management requires specialized medications that are not typically used in primary care settings. Given the intricacies of PAH and its inherently multidisciplinary nature, it is crucial for patients to undergo an assessment at a specialized PAH referral center, in which pulmonologists, cardiologists, rheumatologists, gastroenterologists, and other relevant specialists work in close collaboration. This approach ensures accurate diagnosis, determination of the specific etiology, proper risk stratification, and optimal therapeutic decision-making.

Brazil currently lacks standardized criteria for designating PH referral centers. To address this gap, the following recommendations are proposed: (1) multidisciplinary expertise: centers should employ a diverse team of specialists proficient in PH management, including pulmonologists, cardiologists, rheumatologists, radiologists and other relevant medical and non-medical specialists; (2) diagnostic capabilities: centers must have access to essential diagnostic tools, RHC being mandatory; (3) treatment resources: a full range of available therapies should be accessible; (4) patient volume: centers should regularly receive new patients with suspected or confirmed PAH and maintain a minimum number of patients under ongoing care; and (5) quality assurance: centers are expected to maintain comprehensive patient records, evaluate and report treatment outcomes, and, ideally, participate in ongoing and new clinical trials.

## FINAL CONSIDERATIONS AND FUTURE DIRECTIONS

The diagnostic workup investigation of PAH involves a direct search for potential clinical disorders that might be related to its genesis. The accurate diagnosis of PAH requires RHC, which additionally allows the assessment of the severity of the disease. PAH risk stratification should be multiparametric for proper PAH management strategy. These recommendations are summarized in Chart 5, which highlights the main diagnostic pillars currently endorsed for PAH assessment. 

**Chart 5:** Main recommendations for the diagnosis of pulmonary arterial hypertension.

• The clinical presentation of pulmonary arterial hypertension is not disease specific, and its diagnostic investigation requires a direct search for clinical conditions that may be associated with its genesis
• Echocardiography is the most important noninvasive screening tool for pulmonary hypertension, detecting indirect signs of increased pulmonary pressure based on the velocity of tricuspid regurgitation jet, as well as providing information on right heart size and function
• Right heart catheterization is the gold standard procedure for the diagnosis and the hemodynamic classification of pulmonary hypertension and should be performed in pulmonary hypertension expert centers
• Right heart catheterization is needed to assess the severity of pulmonary vascular and right ventricular impairment and to perform vasoreactivity testing of the pulmonary circulation, when appropriate
• Vasoreactivity testing should only be performed in idiopathic, hereditary, or drug-induced pulmonary arterial hypertension.
• Hemodynamic fluid challenge should be considered to uncover a post-capillary hemodynamic pattern in patients suspected of having occult left heart disease
• Exercise right heart catheterization should be considered for the investigation of dyspnea of unknown origin, when resting hemodynamics are normal, or to access functional status, response to treatment, and prognosis in patients with established pulmonary hypertension
• Pulmonary arterial hypertension risk stratification is essential for therapeutic planning, at the time of diagnosis and during patient follow-up, and should be multiparametric for proper pulmonary arterial hypertension management strategy

Advances in genetic testing and biomarker research are paving the way for more personalized approaches to the diagnosis and management of PAH. As our understanding of the complex comorbidities and heterogeneity among PAH patients grows, integrating genetic profiles, emerging biomarkers, and multimodality imaging techniques into clinical practice could enable better tailored treatment strategies, ultimately improving outcomes through precision medicine in the future.
